# Sturge-Weber Syndrome With Leptomeningeal Angiomatosis and Middle Cerebral Artery Narrowing Treated With Intravenous Tissue Plasminogen Activator (IV tPA): A Case Report

**DOI:** 10.7759/cureus.105299

**Published:** 2026-03-16

**Authors:** Gurleen Kaur, Sahil Sardana, Nihas R Mateti, Jaylene Debiec, Muhammad Saim, Hassan Abdullah, Romil Singh, David Wright, Russell Cerejo

**Affiliations:** 1 Neurology, University at Buffalo Jacobs School of Medicine and Biomedical Sciences, Buffalo, USA; 2 Neurology, Allegheny Health Network, Pittsburgh, USA; 3 Medicine, Osmania Medical College, Hyderabad, IND; 4 Neurology, Nishtar Medical University, Multan, PAK

**Keywords:** mca, middle cerebral artery (mca), stroke, sturge weber syndrome, tenecteplase (tnk)

## Abstract

Sturge-Weber syndrome (SWS) is a rare and congenitally acquired neurocutaneous disorder characterized by port wine stain, leptomeningeal angiomas with or without glaucoma. Clinical presentation includes focal motor epilepsy as the first symptom. They may also develop migraines, cognitive impairment, blindness secondary to glaucoma, and transient stroke-like episodes. We report a case of a 60-year-old patient with SWS with known leptomeningeal angiomatosis presenting with aphasia in the setting of left middle cerebral artery narrowing who received intravenous tissue plasminogen activator (IV tPA). Neurocutaneous syndromes associated with intracranial vascular malformations have been considered at a higher risk of intracranial bleeding following IV thrombolytic therapy. We report the first case to our knowledge in which a patient with SWS with known leptomeningeal angiomatosis received IV tPA for acute stroke therapy without intracranial bleeding.

## Introduction

Sturge-Weber syndrome (SWS) is a rare sporadic congenital neurocutaneous disorder caused by a somatic mosaic mutation of the GNAQ gene. It occurs at an estimated frequency of between 1:20,000 and 1:50,000. Sporadic mosaic mutation in the GNAQ gene has been associated with SWS and non-syndromic port wine stain [1]. In total, encephalofacial angiomatosis has been classified into three types; SWS falls into type 1, which includes both facial and leptomeningeal angiomas, with or without glaucoma. Eight-five percent of cases of brain angiomas are ipsilateral to the side of the port wine stain; no relationship has been noted between the extent of the facial lesion and the severity of the brain lesion [2]. Very often, focal motor epilepsy is the first presenting feature; about 15% of the patients with bilateral intracranial involvement are associated with a higher incidence of seizures, with outcomes being poor in those patients. One-third of the patients would have seizures coinciding with febrile illness [3]. Migraines, cognitive impairment, hemiplegic migraine, and transient stroke-like episodes have also been associated with SWS in the literature, with symptomatic treatment [1]. To our knowledge, there are only seven reported cases in all different age groups to date in patients with SWS who developed spontaneous intracranial hemorrhages [4], leaving us with no calculated incidence rate. The association of SWS with ischemic or hemorrhagic strokes is debatable, given that it is tough to differentiate between hemiplegic migraine, postictal state, and actual ischemic event in SWS.

## Case presentation

**Figure 1:**
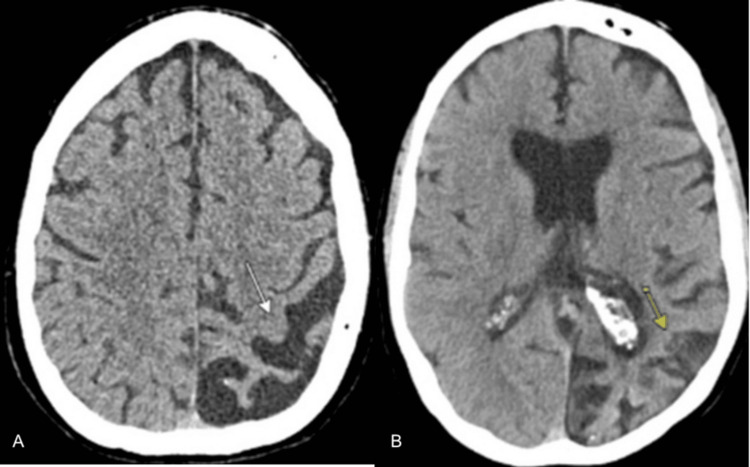
(A, B) Left parietal lobe cerebral brain volume loss due to sequelae of chronic ischemia secondary to SWS (arrows) SWS: Sturge-Weber syndrome

**Figure 2:**
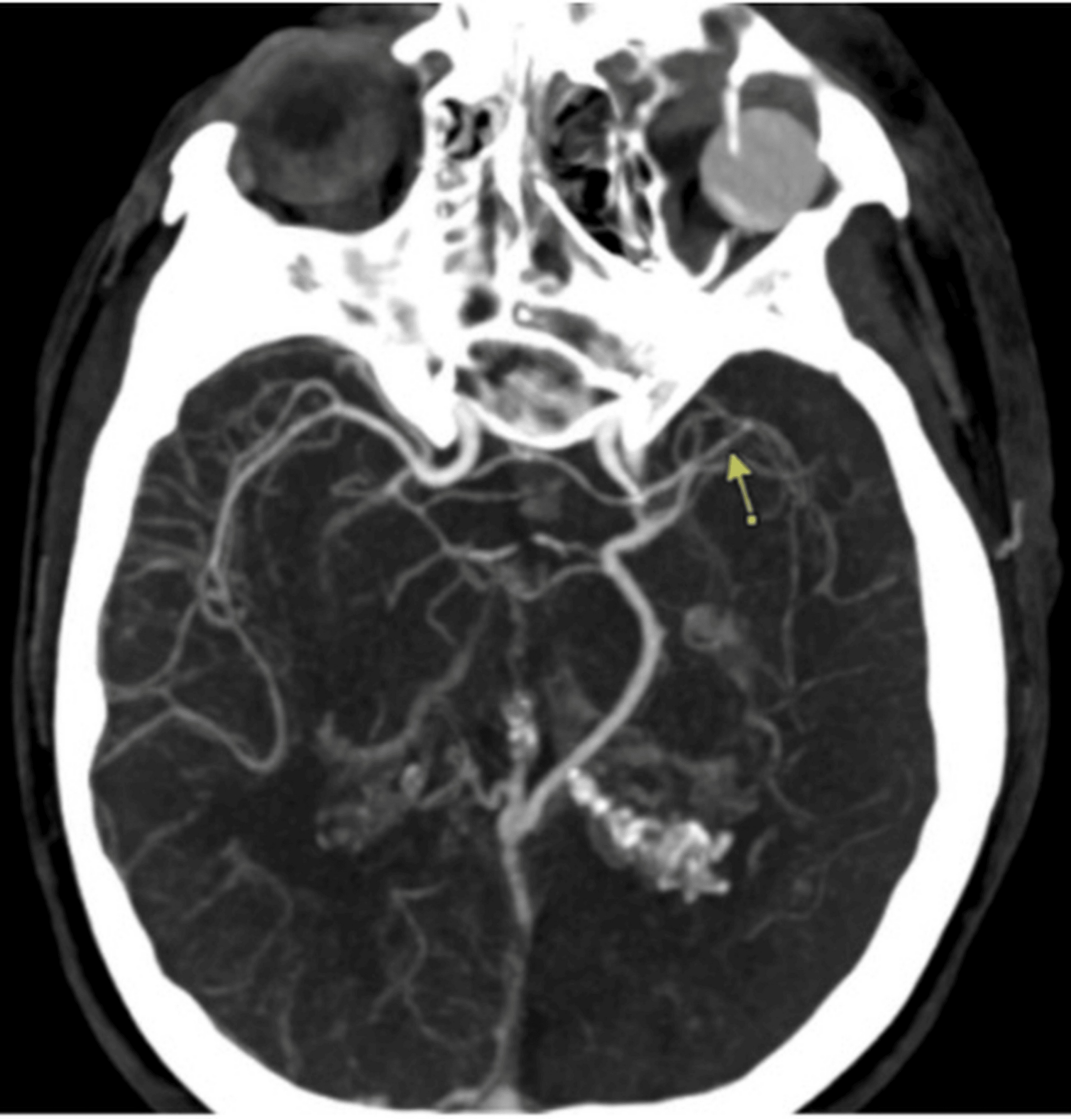
CT angiogram of the head showing new moderate-to-severe diffuse narrowing of the left middle cerebral artery (arrow)

A 60-year-old female presented to our institution with an acute onset of aphasia within two hours of her last known well. She had a history of SWS with left facial port wine stain, left eye blindness due to glaucoma, left occipital leptomeningeal angiomatosis, migraine, epilepsy (diagnosed at 58 years of age), coronary artery disease, and prior tobacco use. The patient's presenting blood pressure was 160/90. The patient initially had severe pain in her right arm and leg with spasms, which resolved spontaneously within minutes. She then had a sudden onset of aphasia. In the emergency room, her National Institutes of Health Stroke Scale was 6 for severe aphasia (both expressive and receptive), not following commands, and not answering level of consciousness questions (disoriented to time and place) correctly. Noncontrast CT head showed left parietal lobe cerebral brain volume loss due to sequelae of chronic ischemia with an Alberta Stroke Program Early CT Score (ASPECTS) of 8, but no other acute findings (Figure 1). CT angiogram of the head and neck showed new moderate-to-severe diffuse narrowing of the left middle cerebral and posterior cerebral arteries compared with the CT angiogram two years ago (Figure 2). The potential risk of hemorrhage was discussed with the patient’s family as part of the shared decision-making process prior to treatment. The decision was made to proceed with intravenous tissue plasminogen activator (tPA) administration, as the patient was within the 4.5-hour window from symptom onset. She did have a history of epilepsy, but she was found unresponsive during those episodes, and there was no clear record of her seizures presenting as aphasia alone. Also, her presentation was clear and acute in onset, without any witnessed seizure-like activity. She also did not complain of any headache to the emergency medical services (EMS) prior to the onset of aphasia, making stroke mimics less likely in the differentials. She had a 20-minute EEG done in the emergency room that showed no seizures. Later, an MRI of the brain was performed, which demonstrated no diffusion restriction or hemorrhage. The CT head performed 24 hours after tPA administration did not show any evidence of hemorrhage. Her symptoms lasted about 24 hours and completely resolved during her three-day hospital stay, and she was discharged home.

## Discussion

Defining an underlying etiology in SWS patients presenting with stroke-like symptoms is arduous. As our patient was fully independent at baseline and had disabling symptoms with a new left middle cerebral artery (MCA) branch stenosis, IV thrombolysis was considered. She did not have any clear documentation of her seizure semiology, as they were mostly unwitnessed events; however, aphasia was the sole symptom.

The new moderate-to-severe narrowing of the left MCA in our patient may reflect the dynamic vasculopathy described in SWS, further complicating diagnostic interpretation. Recent neuroimaging literature has emphasized the role of chronic venous hypertension, impaired autoregulation, and regional perfusion abnormalities in the pathophysiology of stroke-like episodes in SWS, reinforcing the biological plausibility of transient ischemia in these patients [5].

To our knowledge, there is only one case report of tPA administration in an SWS patient with pulmonary embolism by George et al., in which no intracranial bleeding was reported [6]. A case report by Aguglia et al. reported a case with a temporal lobe hemorrhage in a patient with SWS. They emphasized the importance of obtaining CT angiography in SWS patients to detect MRI-silent angiomatosis, even when MRI is unremarkable [7]. There is limited data on tPA administration in patients with intracranial vascular malformations or neurocutaneous syndromes. Recent consensus recommendations indicate that the risk-benefit profile of intravenous thrombolysis in patients with unruptured intracranial vascular malformations remains uncertain; however, tPA may be considered in cases of severe or potentially disabling ischemic stroke when the anticipated benefit outweighs the theoretical hemorrhagic risk. These recommendations emphasize individualized clinical judgment rather than automatic exclusion based solely on the presence of a vascular lesion [8].

In a study done by Erdur et al. of 350 patients who received IV tPA, one out of nine patients with cavernous cerebral malformations (CCM) had a symptomatic intracerebral hemorrhage versus 11 out of 341 patients without CCM who had a symptomatic bleed [9]. Another reported case of a patient with tuberous sclerosis with left MCA syndrome who received IV tPA showed significant improvement in symptoms with no intracerebral hemorrhage [10]. The use of IV tPA for acute ischemic stroke treatment in other cerebral vascular malformation etiologies has also been limited to a few reported cases. Henninger et al. reported the first known case of tPA use in a 79-year-old male with a cavernous malformation. This case reported tPA use within 2.3 hours of symptoms onset, and the patient experienced no intracranial hemorrhage or expansion of cavernous malformation [11]. Theoretically, patients with intracranial vascular malformations may be presumed to carry a higher risk of intracranial hemorrhage following thrombolytic therapy compared with the general population. However, available clinical data do not clearly demonstrate a substantially increased risk. Stone et al. performed a retrospective chart review evaluating thrombolysis outcomes in patients with preexisting vascular malformations, in which 438 patients received IV tPA, and 56 were found to harbor vascular malformations at the time of treatment. Only one patient experienced hemorrhagic transformation, which was attributed to uncontrolled blood pressure and peri-procedural factors rather than the vascular lesion itself, suggesting that thrombolysis may not confer a markedly elevated hemorrhagic risk in carefully selected patients [12].

Current American Heart Association guidelines similarly recognize the presence of an unruptured intracranial vascular malformation as a relative contraindication rather than an absolute exclusion for intravenous thrombolysis, noting that the safety of thrombolysis in this population remains uncertain. This classification supports individualized risk-benefit assessment in patients with potentially disabling deficits, as was undertaken in our case [13].

## Conclusions

The presence of intracranial vascular malformations in patients with SWS introduces uncertainty regarding hemorrhagic risk following intravenous thrombolysis. However, available literature, including observational data from other vascular malformation populations and contemporary guideline recommendations, does not support automatic exclusion from thrombolytic therapy. In carefully selected patients presenting with potentially disabling neurological deficits and no absolute contraindications, individualized risk-benefit assessment may support the use of intravenous thrombolysis. Our case demonstrates that intravenous alteplase administration in a patient with known leptomeningeal angiomatosis and suspected acute ischemic stroke was not associated with intracranial hemorrhage and resulted in complete neurological recovery. The absence of diffusion-restricted infarction on MRI despite disabling symptoms may reflect transient ischemia, dynamic hypoperfusion related to underlying SWS vasculopathy, or a stroke mimic such as peri-ictal aphasia. Importantly, observational studies suggest that patients ultimately diagnosed with stroke mimics have a low risk of symptomatic intracranial hemorrhage following thrombolysis, supporting treatment when disabling deficits are present, and ischemia cannot be confidently excluded in the hyperacute setting. Larger observational studies are needed to better define the safety profile of thrombolysis in patients with neurocutaneous syndromes and intracranial vascular malformations.
